# The diversity of energy consumption structure, energy efficiency and carbon emissions: Evidence from Shaanxi, China

**DOI:** 10.1371/journal.pone.0285738

**Published:** 2023-05-17

**Authors:** Tengfei Yin

**Affiliations:** School of Management, Xi’an University of Finance and Economics, Xi’an, China; Shenzhen University, CHINA

## Abstract

As a major energy sources province in China, Shaanxi Province ranks top three in terms of raw coal production in China and undertakes the important task of ensuring national energy supply and security. Affected by the endowment of energy resources, fossil energy accounts for a large proportion of the energy consumption structure in Shaanxi Province, and it will face huge challenges under the severe carbon emission situation in future. In order to analyze the relationship between energy consumption structure, energy efficiency and carbon emissions, the paper introduces the concept of biodiversity into the energy industry. Taking Shaanxi Province as an example, the paper calculates the energy consumption structure diversity index and analyzes the impact of energy consumption structure diversity on energy efficiency and carbon emissions in Shaanxi Province. The results shows that the diversity index and equilibrium index of energy consumption structure in Shaanxi exhibits a slow upward trend in general. In most years, energy consumption structure diversity index in Shaanxi is higher than 0.8, and the equilibrium index is higher than 0.6. The carbon emissions of energy consumption in Shaanxi generally show increasing trend, and the carbon emissions have increased from 5,064.6 tons to 21899.67 tons from 2000 to 2020. The paper also shows that Shaanxi H index is negatively correlated with total factor energy utilization efficiency in Shaanxi, and positively correlated with carbon emissions in Shaanxi. The main reason is the internal substitution of fossil energy, and the proportion of primary electricity and other energy sources is still relatively low, which leads to a higher level of carbon emissions.

## Introduction

The energy consumption structure is closely related to economic development [1], energy intensity [2], total energy consumption [3, 4], industrial structure [5], and preference for energy consumption demand guided by energy policies [6–9]. Scholars have carried out a lot of research using the grey relational prediction method [10], input-output method [11], mechanism design methodology [12], index decomposition method [11, 13], Divisia agglomeration analysis method [14], RAS model [15], LMDI model [16–18] and other methods to study the influence factors of energy consumption, the trend of total energy consumption, and the optimization path of energy consumption structure, the relationship between energy consumption structure and energy intensity. The research conclusions are mainly used for the adjustment and optimization of the energy consumption structure [2], including the development of the tertiary industry, the control of high energy consuming industries, the cultivation of emerging low-carbon industries, the integration of coal resources, the implementation of energy price mechanism reform, the promotion of technological progress, cost sharing, the adjustment of energy consumption in the private sector, the guidance of residents’ energy consumption preferences, and the substitution of capital energy etc.

The above energy structure adjustment strategies have been partially applied to the development of Chinese energy industry policies, and a series of policies have been published to encourage the development of new energy industries and restrict the expansion of high energy-consuming industries, the main purpose is to improve the energy consumption structure through continuous industrial structure optimization. After nearly 6 years of supply-side structural reforms in the coal industry, energy consumption structure in China has been optimized, the proportion of coal consumption continues to decline, and the proportion of new energy and renewable energy consumption increases gradually. The energy consumption structure is more diversified, forming an energy production and consumption system of coal as the mainstay paralleling development with oil, natural gas, hydropower, wind power, solar power, nuclear power, etc. It is foreseeable that with the optimization of China’s industrial structure, the improvement of new energy investment capacity, and the improvement of the profitability of renewable energy, energy consumption diversity will become an important development direction of China’s energy industry. So how to establish the energy consumption diversity evaluation index, how to determine energy consumption diversity level of Shaanxi Province, and how to understand the current situation and evolution trends of energy consumption structure diversity, are all important issues to be faced after the adjustment of energy structure.

In the evaluation of energy diversity, some scholars have discussed the evaluation of energy diversity and its impact on energy security and energy utilization efficiency from the perspective of energy diversity. The Shannon -Wiener diversity index was used to calculate the diversity of primary energy supply in China from 1949 to 2005 [19] and obtained the evolution rule of energy diversity based on the proportions of coal, crude oil, natural gas, hydropower, and nuclear power, and analyzed the impact of structural changes on energy diversity [20, 21].

An energy diversity index system is established, including the diversity of domestic energy supply and energy import, and proposes that energy diversity is one dimension to measure energy security [22]. A cointegration analysis model between energy structure diversity and energy efficiency is also established based on the data of 30 provinces, municipalities, and autonomous regions in China, the research shows that there is a cointegration relationship between energy structure diversity and energy efficiency, and the energy efficiency will increase by 12.47% with the energy diversity index increasing by one unit [23]. Diversity index is a concept in biology and one evaluation dimension of many systems [24, 25]. In recent years, some scholars have introduced the concept of diversity into the energy structure and established an evaluation method for energy structure diversity [26], which provides a fresh perspective for studying the optimization of energy structure [27]. With the development of China’s economy, the contradiction between energy demand growth and environmental protection has become increasingly prominent. Increasing energy consumption diversity is an effective way to alleviate the issue. After more than 3 years of implementation of the coal overcapacity policy, the replacement of coal with new energy and renewable energy has become a strategic for the development of China’s energy industry. Diversified energy supply and consumption systems are gradually being formed, and there are more and more types of energy consumption. The characteristics of diversified energy consumption will have a significant impact on energy utilization efficiency and carbon emissions. Therefore, it is an urgent to establish an index system for evaluating energy consumption structure diversity, and deeply explore the mechanism of energy diversity on energy utilization efficiency and carbon emissions under special situation of energy diversity in China now.

The concept of diversity is original from ecology, organisms are affected by factors such as genes, populations, species, and ecosystems, and often show multiple types and levels in the process of evolution, which makes the ecosystem show diversity and variability [28]. Biodiversity generally includes genetic diversity, species diversity and ecosystem diversity. Biologists generally believe that biodiversity is the result of the evolution of life for hundreds of millions of years on the earth, and it is also the material guarantee for maintaining the basic functions of organisms and the survival and development of human beings [29]. The core of biodiversity is species diversity, including two concepts of species richness and evenness. Species richness describes the sum of all species in a certain space and belongs to a total dimension. Species evenness describes the uniformity of the number distribution of these species in a certain space, which belongs to another balance dimension. Later, scholars introduce the concept of diversity into all aspects of cultural and society, and gradually formed concepts such as cultural diversity, ecological environment diversity, organizational diversity, and business diversity.

Shannon-Wiener diversity index can measure the level of species diversity [30]. The energy system also plays an important role in the development of human society, and it is also applied to diversity concept, because it is the key driving force and material basis for promoting economic and social development. Therefore, it is appropriate to introduce the concept of diversity into the energy system [31], including energy supply diversity, energy consumption diversity, and energy import diversity [20]. Among them, the energy consumption structure diversity is particularly reflected in the energy end-use consumption which heavily impacts energy efficiency and carbon emissions [32].

Scholar’s research conclusion on the influencing factors of carbon emissions can be summarized into four aspects. From the perspective of economic factors, the main factors affecting carbon emissions include economic growth, trade openness, industrial level, energy consumption and so on [33]. From the perspective of institutional policy, financial system and regulatory regulations also have an important impact on carbon emissions [34]. From the perspective of energy system, coal, oil and other fossil fuels are crucial to carbon emissions [35]. From the perspective of urban development, urbanization will aggravate carbon dioxide emissions [36]. However, these research perspectives are mostly focused on economic development and market regulation, the mechanism of carbon emission within the energy system has not been carefully explored.

Discussing the diversity of energy consumption structure will provide an interesting new way of thinking for carbon emission reduction. As a large province of energy supply, Shaanxi’s energy consumption structure has attracted much attention, what impact does it have on local energy efficiency and carbon emissions? In this paper, the diversity calculation method will be used to calculate the energy consumption structure of Shaanxi, China, and establish a regression model between the energy consumption structure, energy efficiency and carbon emissions, and discuss the relationship between them.

The structure of this paper is as follows. First of all, based on the basic data of energy consumption in Shaanxi Province, using the Shannon-Wiener diversity index to calculate the diversity index of energy consumption structure in Shaanxi Province, and analyze the calculation results. Secondly, the emission factor method is used to estimate the carbon emissions of Shaanxi’s energy consumption, and the DEA is used to measure the energy utilization efficiency. Finally, the paper constructs the regression model between the diversity of energy consumption structure and energy efficiency carbon emissions, and discusses the impact of the diversity of energy consumption structure on energy efficiency and carbon emissions.

## Materials and methods

### Energy consumption structure diversity index

The abbreviations referred to in the paper are as follows. H index stands for diversity index, E index stands for equilibrium index, DEA stands for Data Envelopment Analysis, DMU stands for Decision Making Units.

Reference to the concept of species diversity, the paper defines the energy consumption structure diversity as: within a certain period, the degree of heterogeneity and equilibrium of various energy sources in energy consumption, including the richness of energy consumption and the degree of equilibrium of energy consumption. The richness of energy consumption refers to the number of various energy types in the process of energy consumption, and the equilibrium degree of energy consumption refers to the proximity of various energy consumption in the process of energy consumption. The energy consumption structure diversity index is:

$$H=-\overset{N}{\underset{i=1}{\sum }}P_{i}lnP_{i}$$ (1)

Among them, H represents the energy consumption structure diversity, N represents the number of energy types in the primary energy consumption structure, and *P*_*i*_ represents the proportion of the energy consumption in the primary energy consumption structure, which essentially reflects the weighted geometric mean of the proportion of each energy consumption in the primary energy consumption structure. The value range of H is greater than or equal to 0, if there is only one energy source in the primary energy consumption structure, then *P*_*i*_ equal to 1, then the value of H is equal to 0. If the energy types in the primary energy consumption structure increases, the value of H also increases accordingly. The value of H is related to the type of energy consumption and the proportion of each energy consumption. If the number of energy types is given, when the value of H increase, it means that the consumption of various energy sources tends to be equal in the primary energy consumption and the types of energy consumption are more. And when the value of H value decreases, it means that the primary energy consumption mainly depends on one or several energy sources, the proportion of consumption of different energy sources may be expanding.

The energy consumption structure equilibrium index is:

$$E=\frac{H}{H_{max}}$$ (2)

Among them, *H*_*max*_ represents the maximum value of the energy consumption structure diversity, and in the maximum equilibrium state, the maximum value of the diversity index is *lnN*. It can be seen from the formula that when the energy consumption structure equilibrium index E approaches to 0, it means that the non-equilibrium degree of the primary energy consumption structure increases; when the energy consumption structure equilibrium index E approaches to 1, it means that each energy types in primary energy consumption structure is nearly equal.

The Shannon-Wiener Diversity Index and Equilibrium Index formulas are used to calculate the changes in the energy consumption structure diversity in Shaanxi Province from 2000 to 2021. The data comes from the "Shaanxi Statistical Yearbook 2001–2022". The official data released by the Bureau of Statistics shows that the primary energy consumption in energy consumption statistics is mainly divided into four types: coal, oil, natural gas, primary electricity and other energy. The total energy consumption and consumption structure in Shaanxi, as well as the results of the measured diversity index H and equilibrium index E are shown in Table 1.

**Table 1:** Shaanxi’s total energy consumption and energy consumption structure.

years	Total energy consumption/10,000 tons of standard coal	Proportion of Total Energy Consumption				H-index	E-index
		Coal	Oil	Natural gas	Primary electricity and other energy		
2000	2616.78	0.7130	0.2330	0.0310	0.0230	0.7751	0.5591
2001	3034.34	0.6973	0.2426	0.0434	0.0167	0.7995	0.5767
2002	3447.88	0.6890	0.2481	0.0493	0.0136	0.8093	0.5838
2003	3918.96	0.7110	0.2290	0.0560	0.0040	0.7636	0.5508
2004	4692.66	0.6904	0.2201	0.0848	0.0046	0.8229	0.5936
2005	5571.34	0.7560	0.1740	0.0410	0.0300	0.7519	0.5424
2006	6129.36	0.7520	0.1660	0.0620	0.0210	0.7660	0.5525
2007	6774.86	0.7400	0.1560	0.0790	0.0250	0.8054	0.5810
2008	7417.46	0.7010	0.1830	0.0930	0.0230	0.8675	0.6257
2009	8043.60	0.7150	0.1750	0.0830	0.0270	0.8490	0.6124
2010	8287.63	0.7053	0.1715	0.0896	0.0337	0.8790	0.6341
2011	9107.48	0.7206	0.1586	0.0901	0.0308	0.8522	0.6147
2012	9914.53	0.7319	0.1585	0.0812	0.0285	0.8257	0.5956
2013	10610.48	0.7230	0.1558	0.0855	0.0357	0.8534	0.6156
2014	11222.46	0.7241	0.1506	0.0890	0.0363	0.8545	0.6164
2015	11745.93	0.7262	0.1217	0.1050	0.0471	0.8692	0.6270
2016	12146.47	0.7497	0.0961	0.1051	0.0491	0.8258	0.5957
2017	12548.52	0.7466	0.0860	0.1066	0.0608	0.8381	0.6045
2018	12900.38	0.7378	0.0840	0.1058	0.0724	0.8602	0.6205
2019	13478.06	0.7273	0.0772	0.1143	0.0813	0.8813	0.6357
2020	13512.26	0.7526	0.0637	0.1046	0.0791	0.8261	0.5959
2021	14515.23	0.7369	0.6000	0.1040	0.0991	0.8583	0.6191

**Fig 1:**
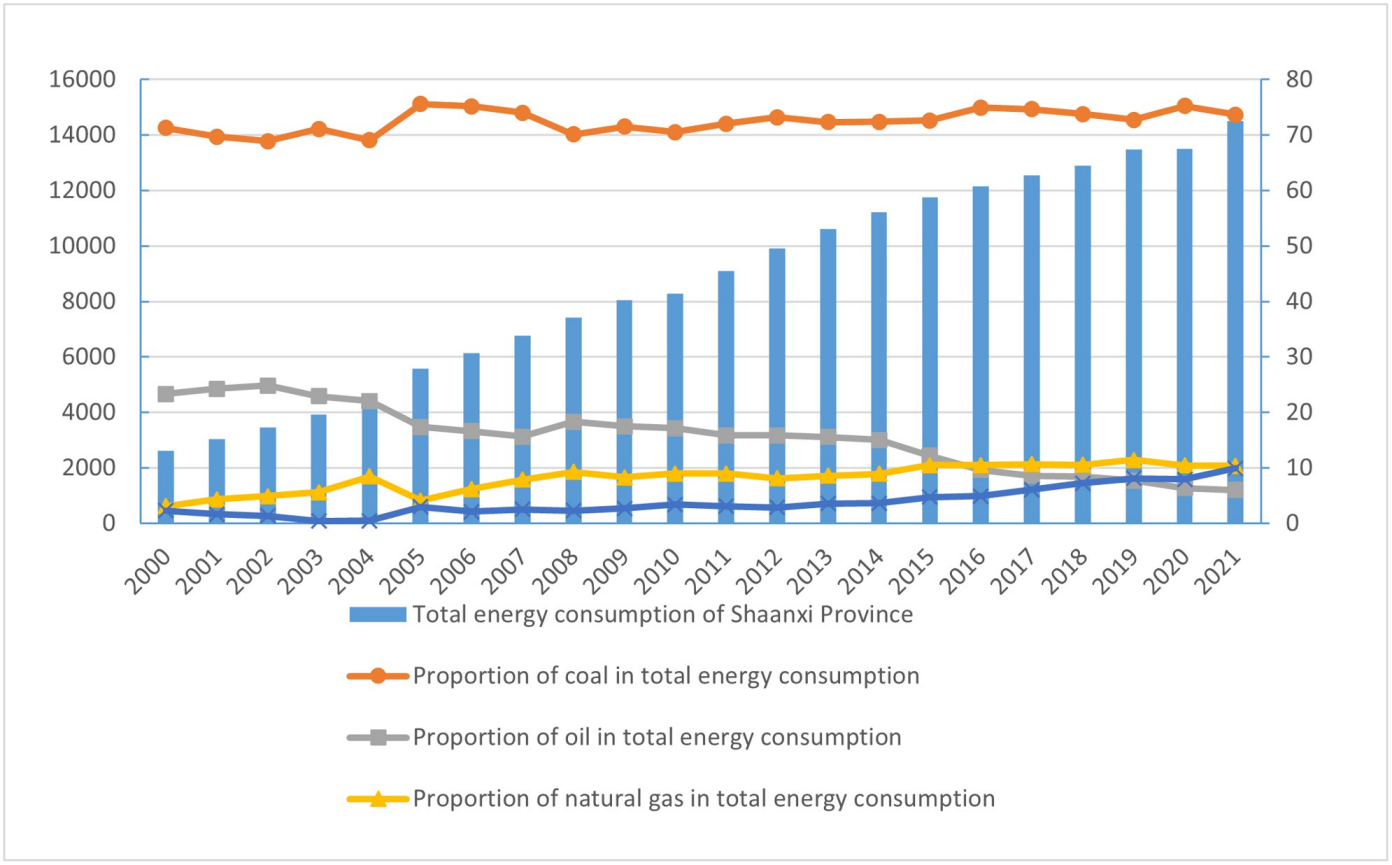
The trend of total energy consumption and energy consumption structure in Shaanxi.

The changing trend of total energy consumption and energy consumption structure in Shaanxi is shown in Fig 1. First, from the perspective of total energy consumption, the total energy consumption in Shaanxi shows a steady increasing overall from 2000 to 2021, and the total energy consumption increases from 26.1678 million tons of standard coal in 2000 to 145.1523 million tons in 2021, the continuous increase of Shaanxi’s total energy consumption is mainly driven by the energy demand and industrial structure caused by Shaanxi’s economic growth. On the one hand, Shaanxi’s economic growth rate is relatively high, and the average growth rate of Shaanxi’s GDP is 10.0% from 1979 to 2020, 0.8 percentage points higher than the national GDP growth rate over the same period. On the other hand, the high energy consuming industries in Shaanxi have a relatively strong demand for energy, including coal, petroleum and other fuel processing industries, chemical raw material and product manufacturing, non-metallic mineral products, ferrous metal smelting and processing, and non-ferrous metal smelting and processing industries, electricity, heat, gas and water production and supply industries. In 2021, the total energy consumption of above six industries accounts for 87% of the industrial energy consumption above designated size, with a year-on-year increase of 10.8%. Second, from the perspective of energy consumption structure, from 2000 to 2021, Shaanxi’s energy consumption structure shows the characteristics of " two liters, one down and one unchanged ", Specifically, the proportion of natural gas, primary electricity and other energy in the total energy consumption of Shaanxi shows a slowly rising trend, the proportion of oil in Shaanxi’s total energy consumption generally shows a volatile downward trend, and the proportion of coal in Shaanxi’s total energy consumption is generally unchanged. Although there are fluctuations in individual years, coal still accounts for 70% of the total energy consumption in Shaanxi. In 2021, Shaanxi’s coal consumption accounts for about 73.69% of the total energy consumption, which is much higher than the national level of 56% in the same period. It can be seen that the optimization of Shaanxi’s energy structure still faces severe challenges. At the same time, we should pay more attention that the internal substitution effect of Shaanxi’s energy structure is gradually emerging. Natural gas, primary electricity and other energy sources gradually replace oil and become an important role in energy supply. In 2021, natural gas, primary electricity and other energy sources accounts for 10.40% and 9.91% of Shaanxi’s total energy consumption, higher than 6% of the oil proportion of. Among them, primary power and other energy sources include solar power, hydropower, wind power, nuclear power and other renewable energy sources, which is also an important achievement of Shaanxi’s active promotion of new energy and clean energy development strategies.

**Fig 2:**
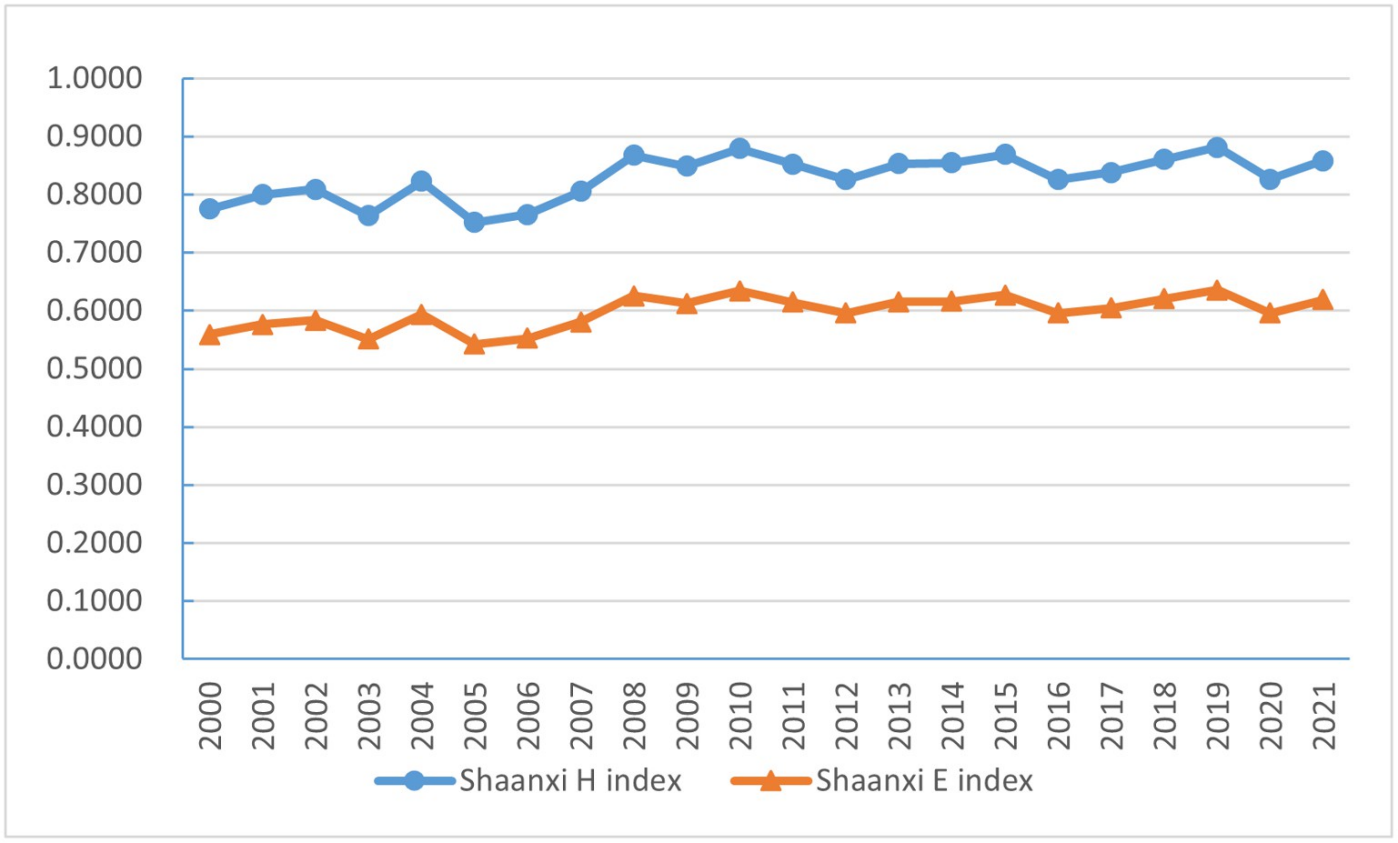
The diversity index of energy consumption structure in Shaanxi.

The diversity index of energy consumption structure in Shaanxi is shown in Fig 2. It can be seen that from 2000 to 2021, the Shaanxi energy consumption structure diversity index and equilibrium index generally shows a slow upward trend, and Shaanxi energy consumption structure diversity index is higher than 0.8 in most years, the equilibrium index is higher than 0.6, but lower than the national energy consumption structure diversity index and equilibrium index in the same period. According to the change of the diversity index, it can be roughly divided into three stages, the first stage is from 2000 to 2005, in this stage, the diversity index and equilibrium index of Shaanxi energy consumption structure show a fluctuating decline, and the energy consumption structure of Shaanxi is diverse. The diversity index dropped from 0.7751 in 2000 to 0.7519 in 2005, and the equilibrium index dropped from 0.5591 in 2000 to 0.5424 in 2005, indicating that Shaanxi’s energy consumption is highly dependent on coal resources at this stage, the gap of various energy consumption within the energy system is widening. The second stage is from 2006 to 2012, in this stage, both the Shaanxi energy consumption structure diversity index and equilibrium index have shown a rapid upward trend. The Shaanxi energy consumption structure diversity index increased from 0.766 in 2006 to 0.8257 in 2012. The equilibrium index increased from 0.5525 in 2006 to 0.5956 in 2012, indicating that the optimization of energy consumption in Shaanxi continued to improve at this stage, and the consumption of natural gas, primary electricity and other energy was increasing, and the equilibrium degree of various energy consumption within the energy system had increased. The third stage is from 2013 to 2021, in this stage, the diversity index and equilibrium index of Shaanxi’s energy consumption structure are basically stable, and the diversity index and equilibrium index are maintained at around 0.8 and 0.6 respectively, which on the one hand reflects that the status quo of high proportion of coal in the energy consumption structure has not been changed, on the other hand, it also reflects that the types of energy sources in Shaanxi have not increased, and the main energy structure is basically stable.

### Carbon emission accounting methods

At present, the main methods of carbon emission accounting are emission factor method, material balance method and actual measurement method. The emission factor method is the main method of IPCC and international carbon emission accounting, which has good applicability and operability. The emission factor method is selected to calculate the carbon emission of energy consumption in Shaanxi. The calculation formula of the emission factor method is:

$$C=\overset{n}{\underset{i=1}{\sum }}E_{i}\times CF_{i}\times CC_{i}\times ROX_{i}\times \gamma$$ (3)

Among them, C represents the carbon dioxide emissions of energy consumption, i represents the number of energy types, *E*_*i*_ represents the consumption of the ith energy, *CF*_*i*_ represents the calorific value of the i_th_ energy, *CC*_*i*_ represents the carbon content of the i_th_ energy, *ROX*_*i*_ represents the carbon oxidation rate of the i_th_ energy, *γ* is a coefficient that represents the ratio of the relative molecular mass of carbon dioxide to the relative atomic mass of carbon, generally taking 3.67. Reference to the research method of Xu Shichun [37], the consumption of fossil energy is used as an accounting indicator, and raw coal, crude oil, natural gas, etc. are selected as carbon emission sources, and the carbon emission value is estimated according to the carbon dioxide emission coefficient of different energy sources.

Shaanxi’s fossil energy consumption includes raw coal, crude oil, and natural gas. The fossil energy consumption data comes from the "Shaanxi Statistical Yearbook 2001–2022". The 2000–2010 Statistical Yearbook did not publish the absolute value of each energy consumption, so the corresponding data is calculated from the ratio of total energy consumption to consumption structure. The carbon emission results calculate by formula (3) are shown in Table 2.

**Table 2:** Consumption structure and carbon emissions in Shaanxi Province.

years	Raw coal/10,000 tons	Crude oil/ton	Natural gas/ton	Carbon emissions/ton
2000	1865.76	609.71	81.12	5064.60
2001	2115.85	736.13	131.69	5949.27
2002	2375.59	855.42	169.98	6806.43
2003	2786.38	897.44	219.46	7699.45
2004	3239.81	1032.85	397.94	9175.06
2005	4211.93	969.41	228.42	10284.96
2006	4609.28	1017.47	380.02	11356.25
2007	5013.40	1056.88	535.21	12419.00
2008	5199.64	1357.40	689.82	13909.78
2009	5751.17	1407.63	667.62	14931.89
2010	5844.95	1421.13	742.26	15260.59
2011	6562.60	1444.03	820.15	16652.86
2012	7255.99	1571.34	804.67	18153.82
2013	7671.85	1652.66	907.10	19268.37
2014	8125.91	1690.24	998.58	20294.32
2015	8530.00	1429.56	1232.92	20592.00
2016	9105.79	1167.72	1276.93	20829.83
2017	9368.33	1078.97	1337.92	21103.85
2018	9518.02	1083.73	1364.83	21413.23
2019	9802.13	1039.89	1540.01	22062.24
2020	10168.92	860.36	1413.60	21899.67
2021	10696.15	871.57	1509.47	22974.54

### Calculation of total factor energy utilization efficiency

Data Envelopment Analysis (DEA) [38], or DEA model for short, is usually used to evaluate the relative effectiveness of multivariate decision making units and judge the relative effectiveness of the decision-making unit according to the deviation degree of the decision making unit from the DEA frontier by controlling input variables and output variables of Decision Making Units (DMU) and using mathematical programming method to project each decision making unit onto the production frontier. The DEA model includes the CCR model and the BCC model, the CCR model evaluates the total efficiency under the condition of constant returns to scale, and the BCC model evaluates pure technical efficiency and scale efficiency under the condition of variable returns to scale.

The DEA model is used to calculate total factor energy utilization efficiency, and the selection of indicators should consider the principles of non-negativity, weak correlation, and controllability. Based on the concept of total factor energy utilization efficiency and research purpose, the paper selects capital stock, labor force and energy consumption as input variables, and the output variables are gross domestic product and carbon emissions.

The estimation method of capital stock K refers to the practice of Allan Young& John C [39]. The perpetual inventory method is used to estimate the capital stock of Shaanxi from 2000 to 2020. In order to exclude the influence of inflation, year 2000 is set as the base period, the paper converts the fixed asset investment into the constant price of the base period of year 2000 by using fixed asset investment price index. The calculation formula of the perpetual inventory method is:

$$K_{t}=K_{t-1}\left(1-\delta_{t}\right)+\frac{I_{t}}{P_{t}}$$ (4)

Among them, *K*_*t*_ represents the capital stock of Shaanxi in period t, *δ*_*t*_ represents the economic depreciation rate, usually taking 9.6%, *I*_*t*_ represents the total investment in fixed assets in Shaanxi in period t, and *P*_*t*_ represents the price index of fixed asset investment in year 2000, the value is set as 100.

The estimation of labor force L should fully consider the factors of human capital and reflect the education level of employees. Therefore, the calculation formula of labor force is:

$$L_{t}=l_{t}\times yr_{t}$$ (5)

Among them, *l*_*t*_ represents the number of employees at the end of the year, *yr*_*t*_ represents the average years of education of the population over the age of 15, the number of employees is from the Shaanxi Statistical Yearbook, and the average number of years of education of the population over the age of 15 in 2010 and 2020 comes from the Sixth and Seventh National Census data, the average years of education of the population over the age of 15 in 2000 is estimated using the years of education method, and different years are set according to different levels of education, 0 years for illiteracy, 3 years for literacy classes, 6 years for primary schools, 9 years of junior high school, 12 years of high school and technical secondary school, 16 years of junior college, undergraduate and postgraduate. It is estimated that the average number of years of education of people over the age of 15 in Shaanxi was 8.04 years in 2000.

Shaanxi energy consumption data comes from Shaanxi Statistical Yearbook. The GDP data of Shaanxi is converted from price index of GDP into the constant price of the base period of year 2000, so as to exclude the influence of inflation. The calculation results of capital stock, labor force, energy consumption, GDP and carbon emissions are shown in Table 3.

**Table 3:** Estimated results of output variables and input variables.

Years	Constant price GDP/100 million yuan	Carbon emissions/ton	Capital stock/100 million yuan	Labor force/10,000 people per year	Energy consumption tons of standard coal
2000	1804.00	5064.60	7458.50	14576.52	2616.78
2001	1831.17	5949.27	7589.75	14587.02	3034.34
2002	1847.23	6806.43	7829.94	15561.70	3447.88
2003	1897.40	7699.45	8346.81	16129.63	3918.96
2004	2038.49	9175.06	8996.19	16630.49	4692.66
2005	2209.52	10284.96	9965.23	17191.20	5571.34
2006	2368.78	11356.25	11386.42	17540.35	6129.36
2007	2562.22	12419.00	13486.67	18044.53	6774.86
2008	2822.01	13909.78	16097.95	18546.74	7417.46
2009	2802.51	14931.89	19958.59	19009.68	8043.60
2010	3028.84	15260.59	24859.53	19496.88	8287.63
2011	3311.77	16652.86	29960.19	19722.15	9107.48
2012	3428.62	18153.82	36570.72	19948.14	9914.53
2013	3489.61	19268.37	44797.43	20126.70	10610.48
2014	3483.65	20294.32	54210.14	20421.72	11222.46
2015	3326.84	20592.00	64066.57	20669.67	11745.93
2016	3293.04	20829.83	73553.82	20898.90	12146.47
2017	3444.15	21103.85	83398.07	21088.89	12548.52
2018	3552.33	21413.23	93067.15	21288.96	12900.38
2019	3610.39	22062.24	101773.54	21499.38	13478.06
2020	3585.90	21899.67	109576.27	21597.30	13512.26

Taking GDP and carbon emissions as output variables, capital stock, labor force, and energy consumption as input variables, DEAP2.1 software is used to calculate the total factor energy utilization efficiency of Shaanxi from 2000 to 2020. The calculation results are shown in Table 4. It can be seen that the total factor energy utilization efficiency in Shaanxi is mainly divided into three situations. First, the comprehensive efficiency is 1, indicating that the input and output in this year are DEA effective, and there is no redundancy or surplus. Second, the overall efficiency is less than 1, but the pure technical efficiency is 1, indicating that the support of technical level on energy utilization efficiency has been effectively played in this year, but the investment scale is too large or too small. Among them, there was decreasing returns to scale in 2010, and the scale of investment should be reduced. From 2015 to 2018 and 2020, there is increasing returns to scale, and the scale of input should be increased to obtain higher output.

**Table 4:** DEA result output.

Years	Comprehensive Efficiency	Pure Technical Efficiency	Scale Efficiency	Increase/decrease/constant	Technical Validity
2000	1	1	1	-	efficient
2001	1	1	1	-	efficient
2002	1	1	1	-	efficient
2003	0.999	1	1	-	invalid
2004	1	1	1	-	efficient
2005	1	1	1	-	efficient
2006	1	1	1	-	efficient
2007	0.995	0.995	1	-	invalid
2008	1	1	1	-	efficient
2009	1	1	1	-	efficient
2010	0.989	0.992	0.997	drs	invalid
2011	1	1	1	-	efficient
2012	1	1	1	-	efficient
2013	1	1	1	-	efficient
2014	1	1	1	-	efficient
2015	0.996	0.997	0.999	irs	invalid
2016	0.990	0.993	0.997	irs	invalid
2017	0.988	0.992	0.996	irs	invalid
2018	0.989	0.991	0.998	irs	invalid
2019	1	1	1	-	efficient
2020	0.989	0.991	0.998	irs	invalid
mean	0.997	0.998	0.999		

## Results and discussion

### The relationship between energy consumption structure diversity and energy efficiency in Shaanxi

In order to verify the relationship between energy consumption structure diversity and energy efficiency in Shaanxi, and to reveal the influence factors behind the changes in carbon emissions, the Shaanxi energy consumption structure diversity index H and the proportion of coal consumption are selected as independent variables, and the total factor energy utilization efficiency of Shaanxi is selected as dependent variables, and build a linear regression model, use SPSS19.0 software to fit the model, the regression results are shown in Table 5.

**Table 5:** Shaanxi energy consumption structure diversity and energy efficiency regression results.

Linear Regression Analysis Results n = 21						
	Unstandardized coefficients	Standardized coefficient	t	P	R^2^	F
	B	Beta				
**constant**	1.115	-	27.061	0.000***^a^	0.313	F = 4.099 P = 0.034**
**Shaanxi H Index**	-0.044	-0.363	-1.83	0.084*		
**Coal proportion**	-0.113	-0.494	-2.491	0.023**		
Dependent Variable: Comprehensive Efficiency						

Note

^a)^: ***, **, * represent the significance levels of 1%, 5%, and 10%, respectively

It can be seen that the fitting results of the linear regression model are basically usable. The comprehensive significance test of the model shows that F is 4.099, P is 0.034, and the coefficient of determination is 0.313, indicating that the overall model passes the test at the 5% significance level. The Shaanxi H index passes the significance test at the 10% significance level, and the estimated coefficient of coal proportion passes the significance test at the 5% significance level, indicating that the Shaanxi H index and the estimated coefficient of coal proportion are significant under the corresponding conditions. Based on the above result, the overall model and the estimated coefficients of the two independent variables have passed the significance test.

From the regression results, the estimated coefficient of Shaanxi H index is -0.044, indicating that Shaanxi H index is negatively correlated with total factor energy utilization efficiency in Shaanxi, and for every 1% increase in Shaanxi H index, total factor energy utilization efficiency decreases by 0.044%, indicating that with the improvement of the energy consumption structure diversity index in Shaanxi, total factor energy utilization efficiency is declining. The internal proportion of the energy consumption structure is constantly optimized with more and more investment which leads to a decline in total factor energy efficiency. Therefore, it is necessary to pay attention to the improvement of total factor energy utilization efficiency while improving the energy consumption structure in order to increase GDP. The proportion of coal is negatively correlated with total factor energy utilization efficiency in Shaanxi, and for every 1% increase in coal proportion, total factor energy utilization efficiency decreases by 0.133%, indicating that with the increase in the proportion of coal consumption in total energy consumption in Shaanxi, total factor energy utilization efficiency declines. Excessive proportion of coal consumption will affect the improvement of total factor energy utilization efficiency. Therefore, it is necessary to gradually reduce the proportion of coal consumption to improve total factor energy utilization efficiency.

### The relationship between the energy consumption structure diversity and carbon emissions in Shaanxi

In order to verify the relationship between the energy consumption structure diversity and carbon emissions in Shaanxi, and to reveal the influence factors behind the changes in carbon emissions, the Shaanxi energy consumption structure diversity index H and the proportion of coal consumption is selected as independent variables, The carbon emissions generated by energy consumption in Shaanxi is selected as the dependent variable, and build a linear regression model, use SPSS19.0 software to fit the model, the regression results are shown in Table 6.

**Table 6:** Shaanxi energy consumption structure diversity and carbon emission regression results.

Linear Regression Analysis Results n = 21						
	Unstandardized coefficients	Standardized coefficient	t	P	R^2^	F
	B	Beta				
**constant**	-13.382	-	-13.753	0.000***^a^	0.927	F = 114.139 P = 0.000***
**Shaanxi H Index**	7.175	0.818	12.631	0.000***		
**Coal proportion**	11.087	0.673	10.393	0.000***		
Dependent variable: min-max normalized_carbon emissions						

Note

^a)^: ***, **, * represent the significance levels of 1%, 5%, and 10%, respectively

It can be seen that the fitting result of the linear regression model is ideal. The overall significance test of the model shows that F is 114.139, P is 0.000, and the coefficient of determination is 0.927, indicating that the overall fitting degree of the model is very good. Both the Shaanxi H index and the estimated coefficient of coal proportion have passed the significance test at the 1% significance level, and the null hypothesis that the coefficient is 0 at the 10% significance level can be rejected, indicating that the Shaanxi H index and the estimated coefficient of coal proportion are significant. Based on above result, the model and the estimated coefficients of the two independent variables have passed the significance test.

From the regression results, the estimated coefficient of Shaanxi H index is 7.175, indicating that Shaanxi H index is positively correlated with Shaanxi carbon emissions, and for every 1% increase in Shaanxi H index, Shaanxi carbon emissions increase by 7.175%, indicating that with the energy consumption structure diversity index in Shaanxi increases, carbon emissions will gradually increase. Although the energy consumption structure diversity in Shaanxi increases, it is mainly due to the internal substitution of fossil energy, and the proportion of primary electricity and other energy sources is still relatively low, resulting in a higher output level of carbon emissions. The proportion of coal is positively correlated with carbon emissions in Shaanxi, and for every 1% increase in the proportion of coal, carbon emissions in Shaanxi increase by 11.087%, indicating that carbon emissions also increase with the increase in the proportion of coal consumption in Shaanxi’s total energy consumption. Coal consumption is one of the main factors causing the increase in carbon emissions. Excessive reliance on coal energy will lead to rising carbon emissions. Therefore, it is necessary to gradually reduce the proportion of coal consumption and pay more attention to primary electricity and other energy sources in the process of optimizing the energy consumption structure to reduce carbon emissions.

## Conclusion

**(i)The energy consumption structure diversity in Shaanxi is lower than the national average**. Shaanxi’s energy consumption structure diversity index and equilibrium index generally show a slow upward trend. In most years, the Shaanxi energy consumption structure diversity index is higher than 0.8, and the equilibrium index is higher than 0.6, but lower than the national energy consumption structure diversity index and equilibrium index in the same period. According to the change of the diversity index, it can be roughly divided into three stages. The first stage is from 2000 to 2005, in this stage, Shaanxi energy consumption structure diversity index and equilibrium index show a fluctuating decrease, and Shaanxi energy consumption structure diverse index dropped from 0.7751 in 2000 to 0.7519 in 2005, and the equilibrium index dropped from 0.5591 in 2000 to 0.5424 in 2005, indicating that Shaanxi’s energy consumption is overly dependent on coal resources at this stage, and the gap of various energy consumption within the energy system is widening. The second stage is from 2006 to 2012, both the Shaanxi energy consumption structure diversity index and equilibrium index have shown a rapid upward trend in this stage. The Shaanxi energy consumption structure diversity index increased from 0.766 in 2006 to 0.8257 in 2012. The equilibrium index increased from 0.5525 in 2006 to 0.5956 in 2012, indicating that the optimization of energy consumption in Shaanxi continues to improve at this stage, and the consumption of natural gas, primary electricity and other energy is expanding. The third stage is from 2013 to 2021, Shaanxi energy consumption structure diversity index and equilibrium index are basically stable in this stage, and the diversity index and equilibrium index are maintained at around 0.8 and 0.6 respectively, which reflects that the status quo of high proportion of coal in the energy consumption structure has not been changed, on the other hand, it also reflects that the types of energy sources in Shaanxi have not increased, and the main energy structure is basically stable.

**(ii)The total carbon emissions from energy consumption in Shaanxi is too large, but the growth rate tends to be stable**. From the perspective of total carbon emissions, the carbon emissions of energy consumption in Shaanxi generally shows an increase trend from 2000 to 2021. The carbon emissions increased from 5,064.6 tons in 2000 to 22974.54 tons in 2021 which mainly due to the increase in fossil energy consumption. From the perspective of the growth rate of carbon emissions, although the total carbon emissions from energy consumption in Shaanxi is relatively high, while the relative growth rate is declining year by year. From 2000 to 2014, the growth rate of carbon emissions of energy consumption in Shaanxi dropped rapidly, and the growth rate of carbon emissions decease from 17% in 2001 to 5.3% in 2014, the growth rate of carbon emissions has basically stabilized at around 1% since 2015, indicating that Shaanxi’ active promotion of energy conservation and emission reduction policies has achieved phased results, and carbon emissions have gradually stabilized. In 2020, carbon emissions decreased by 0.7% month-on-month affected by the external economic environment and the epidemic.

**(iii)Total factor energy utilization efficiency in Shaanxi is relatively high**. The average value of total factor energy utilization efficiency in Shaanxi is 0.997, and the total energy utilization efficiency is relatively high. Specifically, it can be divided into three situations. First, the comprehensive efficiency is 1, indicating that the input and output in this year are DEA effective, and there is no redundancy or surplus. Second, the comprehensive efficiency is less than 1, but the pure technical efficiency is 1, indicating that the support of technical level on energy utilization efficiency has been effectively played in this year, while the investment scale is too large or too small. Among them, there was decreasing returns to scale in 2010, and the scale of investment should be reduced. From 2015 to 2018 and 2020, there was increasing returns to scale, the scale of investment should be increased to obtain higher output.

**(iv)The energy consumption structure diversity in Shaanxi is negatively correlated with energy efficiency**. Shaanxi H index is negatively correlated with total factor energy utilization efficiency in Shaanxi, and for every 1% increase in Shaanxi H index, total factor energy utilization efficiency decreases by 0.044%, indicating that with the improvement of the energy consumption structure diversity in Shaanxi, total factor energy utilization efficiency declines. The internal proportion of the energy consumption structure has constantly optimized with more and more investment, which leads to decline in total factor energy efficiency. Therefore, it is necessary to pay attention to the improvement of total factor energy utilization efficiency while improving the energy consumption structure, so as to promote the GDP. The proportion of coal is negatively correlated with total factor energy utilization efficiency in Shaanxi, and for every 1% increase in coal proportion, total factor energy utilization efficiency decreases by 0.133%, indicating that with the increase in the proportion of coal consumption in total energy consumption in Shaanxi, total factor energy Utilization efficiency declines. High proportion of coal consumption will affect the improvement of total factor energy utilization efficiency. Therefore, it is necessary to improve total factor energy utilization efficiency and gradually reduce the proportion of coal consumption.

**(v)The energy consumption structure diversity in Shaanxi is positively correlated with carbon emissions**. Shaanxi H index is positively correlated with Shaanxi carbon emissions, and for every 1% increase in Shaanxi H index, Shaanxi carbon emissions increase by 7.175%, indicating that with the improvement of the diversity index of energy consumption structure in Shaanxi, carbon emissions will gradually increase. Although the energy consumption structure diversity index in Shaanxi is improving, while it is mainly due to the internal substitution of fossil energy, and the proportion of primary electricity and other energy sources is still relatively low, which leads to a higher output level of carbon emissions. The proportion of coal is positively correlated with carbon emissions in Shaanxi, and for every 1% increase in the proportion of coal, carbon emissions in Shaanxi increase by 11.087%, indicating that with the increase in the proportion of coal consumption in Shaanxi’s total energy consumption, carbon emissions are also increasing. Coal consumption is one of the main factors causing the increase in carbon emissions, excessive reliance on coal energy will lead to increase carbon emissions. Therefore, it is necessary to gradually reduce the proportion of coal consumption and pay more attention to reduce the carbon emissions of primary electricity and other energy sources in the process of optimizing the energy consumption structure.

The relationship between the diversity of energy consumption structure and energy efficiency and carbon emissions was discussed. As scholars have concluded, the factors affecting carbon emissions in the energy system are complex, it is difficult to explain the mechanism of carbon emissions using one or two variables. Due to the lack of basic data, the author used the emission factor method in the process of estimating carbon emissions, and did not consider the impact of combustion equipment renewal and energy quality improvement on carbon emissions, which may lead to larger carbon emissions estimation results. A more ideal method is to use the measured basic data of carbon emission sources to summarize the relevant carbon emissions. Therefore, the urgent task is to build a set of accurate, carbon emissions detection system, to form a more comprehensive, objective, accurate, timely value of carbon emissions, to provide data guarantee for the follow-up study. The diversity of energy system is a field worthy of study, in the face of the future world energy supply changes, improve the diversity of energy system can effectively increase the stability of the energy system, to deal with sudden external changes. However, improving the diversity of energy system is a challenging and attractive project, which requires rebuilding infrastructure from energy production, energy transmission, energy storage, energy use and other links, labor-intensive, time-consuming, and costly, which is bound to be a slow process, but also a promising new path of carbon emission reduction.
